# The choice of the white clover population alters overyielding of mixtures with perennial ryegrass and chicory and underlying processes

**DOI:** 10.1038/s41598-022-05100-6

**Published:** 2022-01-21

**Authors:** Isabelle Nölke, Bettina Tonn, Martin Komainda, Sara Heshmati, Johannes Isselstein

**Affiliations:** 1 Division of Grassland Science, Department of Crop Sciences, University of Göttingen, Von-Siebold-Str. 8, 37075 Göttingen, Germany; 2 Centre of Biodiversity and Sustainable Land Use (CBL), University of Göttingen, Büsgenweg 1, 37077 Göttingen, Germany; 3 Present Address: Department of Livestock Sciences, Research Institute of Organic Agriculture (FiBL), Ackerstrasse 113, 5070 Frick, Switzerland; 4 Present Address: Plant Ecology Group, Institute of Ecology and Evolution, University of Tübingen, Auf der Morgenstelle 5, 72076 Tübingen, Germany

**Keywords:** Agroecology, Biodiversity, Grassland ecology

## Abstract

Legume-based forage plant mixtures are known to increase biomass production over the mixture species grown as pure stands (overyielding), which has partly been attributed to enhanced nitrogen availability by legumes. However, the relative importance of underlying processes of these positive diversity effects and their drivers are not fully understood. Here we assessed if outcome and causes of diversity effects depend on the legume-species genetic identity. Over five years, we cultivated different white clover (*Trifolium repens*) populations, a grass and forb species in pure stands and clover-based mixtures and recorded biomass yield. Complementarity and selection effects of mixtures and relative yields of mixture species were calculated based on both unfertilized and nitrogen-fertilized non-leguminous pure stands. Results showed that the clover population altered the overall strength of diversity effects as well as the direction and magnitude of their temporal trends, at least for the grass component of mixtures. Differences in diversity effects between clover populations diminished when fertilized instead of unfertilized non-leguminous pure stands were considered. Hence, a part of these differences likely results from dissimilar effects of clover populations on nitrogen availability. The findings reveal the possibility to improve overyielding of legume-based forage plant mixtures by decisions on legume-species genetic identity.

## Introduction

Plant diversity has long been known to promote aboveground biomass production in extensively manged grassland communities^1,2^. A positive relationship between plant diversity and productivity has also been found in rather intensively managed grassland communities comprising pure stands or mixtures of only one or few productive species^3,4^. Specifically, it could be observed that mixtures produced greater biomass yield than would be expected based on the weighted average biomass yield for the mixture species grown as pure stands^5,6^. That is to say, there was a positive ‘net biodiversity effect’ (NE) or mixtures were overyielding. This has been attributed to niche partitioning between species (‘complementarity’), beneficial effects of species on others (‘facilitation’) or to the inclusion of productive, competitively superior species (‘selection effect’)^7–9^. Among facilitative processes, the most well-studied is that of nitrogen-fixing legumes on non-legumes via their positive effect on the nitrogen availability in the soil^10,11^.

In the majority of past studies, the presence and proportion of legumes explained at least a part of the positive diversity-productivity relationship^12,13^. Accordingly, in agricultural practice, mixtures of grasses with legumes like white clover (*Trifolium repens*) have gained in significance over the more common grass pure stands^14^. Moreover, incorporating non-leguminous forbs into these mixtures is being considered, particularly due to their deep roots that could allow for greater complementarity in water and nutrient use^15,16^.

To assess whether and to what extent mixture overyielding arises from an overall increased species performance in mixture or from particular well-performing species, NE can be partitioned into the contribution of complementarity (CE) and selection effect (SE)^17^. A positive CE indicates that complementarity in the strict sense, facilitation and/or other positive species interactions outweigh competition and/or other negative species interactions, while a negative CE implies the opposite. By contrast, a positive or negative SE is indicative of differences in productivity and competitive ability between species^18,19^.

One critical point associated with the use of these indices is that they mask the behaviour of individual species^20^. However, which species and how strongly these profit from being cultivated in mixtures (overyield in mixture) is crucial to uncover the underlying processes of mixture overyielding^21^. This especially applies to mixtures including legumes, as legumes are often a main driving force of mixture overyielding^13,22^ but are also prone to competitive replacement by their non-leguminous mixture partners^12,23^. Species overyield in mixture when their corrected relative yields (RY_C_) exceed one, i.e. their observed biomass yield in mixture is higher than expected based on their biomass yield in pure stand after accounting for different sowing proportions^24^. Nonetheless, integrated assessments of overall (e.g. NE) and individual diversity effects (e.g. RY_C_) are still not common for experimental communities close to agriculturally used grassland^25^, especially not so for those including non-leguminous forbs^26^.

In agricultural grassland, maintaining high productivity over the whole cultivation period is of major importance and, hence, some of the above-mentioned studies monitored diversity effects over several years^4,25^. These and other studies produced ambiguous results with mostly increasing NE over time due to a growing strength of CE^22,27^. This was more often the result of a rise in mixture yield than the consequence of a decline in the yield of the corresponding pure stands, as might be expected due to weed or pathogen accumulation^28,29^. However, the relative importance of potentially underlying processes, i.e. gradually increasing soil nitrogen pools due to legume activity, variation of other abiotic factors, as well as the time needed to establish niche differentiation or competitive replacement^27,30,31^, has not received much attention. When diversity effects are assessed, unfertilized pure stands typically serve as a reference. Additionally using nitrogen-fertilized non-leguminous pure stands as the baseline to calculate diversity effects can improve our understanding by approximating the contribution of increased soil nitrogen pools by legumes.

Furthermore, previous work has usually focused on the impact of species or functional-group number and identity^22,32,33^ or of species genotype number^6,34^ on diversity effects and their temporal development. The influence of species genotype identity has, so far, not been studied in grassland communities. However, yield potential, competitive ability or complementarity can also vary intra-specifically, as found for different white clover genotypes^35,36^. Legume-species genotypes might further differ in their facilitative effect due to dissimilar nitrogen fixation capacity^37^. Thus, they might differently affect the yield of their non-leguminous mixture partners and its change over time.

In our study, we aimed to examine the outcome and underlying processes of diversity effects in white-clover-based forage plant mixtures with special emphasis on the impact of the genetic identity of white clover. To this aim a five-year field trail at two contrasting sites was conducted. We cultivated eight genetically differing white clover populations as unfertilized pure stands and mixtures with perennial ryegrass (*Lolium perenne*) and/or chicory (*Cichorium intybus*) as well as perennial ryegrass and chicory as unfertilized and nitrogen-fertilized pure stands. Their annual biomass yield was determined and NE, CE and SE of the mixtures^17^ as well as RY_C_ of each mixture species^24^ were calculated based on both unfertilized and fertilized non-leguminous pure stands.

We hypothesized that (1) diversity effects of mixtures and their component species depend on the white clover population and that (2) the white clover population changes temporal trends in diversity effects. Moreover, we expected that (3) a varying influence of the white clover populations on nitrogen availability explains (1) and (2), which would be apparent from diminished differences between populations when considering fertilized instead of unfertilized non-leguminous reference stands.

## Results

### Diversity effects of mixtures with unfertilized non-leguminous references

Considering unfertilized non-leguminous reference stands, CE and SE depended on white clover population, whereas NE did not (Table 1a). While this population effect was unaffected by experimental year, mixture type or study site, the interaction of experimental year, mixture type and study site itself was affecting NE as well as its components CE and SE.

**Table 1:** Effect of white clover population, experimental year, mixture type and study site on complementarity effect (CE), selection effect (SE) and net biodiversity effect (NE) using (**a**) unfertilized (N0) and (**b**) fertilized (N1) non-leguminous reference stands.

Effect	(a) N0						(b) N1					
	CE		SE		NE		CE		SE		NE	
	*F*	*P*	*F*	*P*	*F*	*P*	*F*	*P*	*F*	*P*	*F*	*P*
Population	4.7	< 0.001	4.4	< 0.001	–	–	2.7	0.012	–	–	–	–
Year	270.4	< 0.001	161.7	< 0.001	139.1	< 0.001	452.0	< 0.001	120.0	< 0.001	487.0	< 0.001
Mixtype	128.2	< 0.001	147.3	< 0.001	31.0	< 0.001	3.9	0.023	60.3	< 0.001	31.0	< 0.001
Site	0.0	0.833	24.4	0.003	2.6	0.159	0.0	0.935	18.9	0.005	2.1	0.195
Year × Mixtype	47.4	< 0.001	45.8	< 0.001	22.1	< 0.001	34.4	< 0.001	49.3	< 0.001	45.0	< 0.001
Year × Site	28.8	< 0.001	26.3	< 0.001	49.7	< 0.001	30.0	< 0.001	21.0	< 0.001	40.2	< 0.001
Mixtype × Site	5.2	0.006	9.6	< 0.001	7.3	< 0.001	1.9	0.160	16.1	< 0.001	5.4	0.005
Year × Mixtype × Site	3.6	< 0.001	9.8	< 0.001	11.4	< 0.001	5.7	< 0.001	3.3	0.001	7.9	< 0.001

*F*- and *P*-values of sequential Wald chi-square tests are given. – Effect not retained in the minimum adequate model.

**Figure 1:**
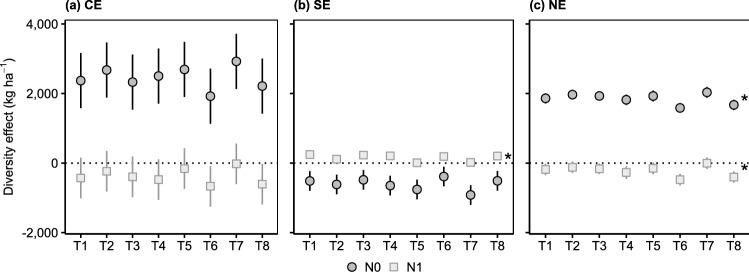
Diversity effects (**a**) complementarity effect (CE), (**b**) selection effect (SE) and (**c**) net biodiversity effect (NE) for different white clover populations (T1–T8) using either unfertilized (N0) or fertilized (N1) non-leguminous pure stands as a reference. Except for *, given values are model predictions with 95% confidence intervals (*n* = 120). Asterisks (*) indicate that arithmetic means with standard errors are shown, as the minimum adequate model did not include the effect of population. Pure stand and mixture yield of the white clover populations can be found in Supplementary Table S4.

The NE and CE of all tested white clover populations were positive, whereas their SE was negative (Fig. 1). Populations with largest positive CE were mostly those with largest negative SE.

**Figure 2:**
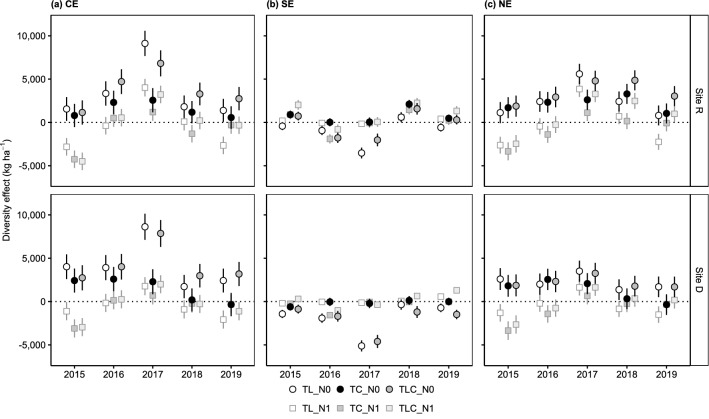
Temporal trends of diversity effects (**a**) complementarity effect (CE), (**b**) selection effect (SE) and (**c**) net biodiversity effect (NE) for different mixture types (TL: clover-grass mixture, TC: clover-chicory mixture, TLC: clover-grass-chicory mixture) at both study sites (site R, site D) using either unfertilized (N0) or fertilized (N1) non-leguminous pure stands as a reference. Given values are model predictions with 95% confidence intervals (*n* = 64). Pure stand and mixture yield of grass, chicory and clover can be found in Supplementary Table S4.

Independent of the white clover population, the clover-chicory mixture was the mixture with the least strong NE, CE and SE at both study sites, but particularly so at site D (Fig. 2). Whereas NE and CE of mixtures including grass were constantly positive and their SE predominantly negative, positive NE and CE of the clover-chicory mixture were restricted to the first experimental years (2015‒2017) and SE of this mixture type was not negative at all (Fig. 2).

In the course of the experiment, NE, CE and SE of mixtures with grass first increased in strength, peaked in 2017, and then decreased at each study site (Fig. 2). By contrast, for the clover-chicory mixture, no (site D) or no pronounced (site R) peaks in NE, CE and SE were found in 2017.

### Diversity effects of mixtures with fertilized non-leguminous references

When using fertilized non-leguminous pure stands as a reference to calculate diversity effects, only CE was altered by white clover population (Table 1b). However, the interaction of experimental year, mixture type and study site was still affecting all studied mixture diversity effects.

Compared to calculations based on unfertilized reference stands, NE, CE and SE were weaker with fertilized reference stands and equalled zero for most white clover populations (Fig. 1). Moreover, populations differed less in their NE, CE and SE with fertilized than with unfertilized references.

The direction of differences in NE and CE between experimental years and mixture types was usually similar between fertilization levels (Fig. 2). Yet, among years, positive NE and CE for all mixture types were only found in 2017. In contrast, SE peaked for neither mixture type in 2017 with fertilized reference stands.

### Diversity effects of mixture species with unfertilized non-leguminous references

With unfertilized non-leguminous pure stands as a reference, white clover population affected grass and clover RY_C_, and for grass RY_C_ this effect further depended on experimental year (Table 2a). In addition, RY_C_ of each mixture species was affected by the interaction between experimental year, mixture type and study site.

**Table 2:** Effect of white clover population, experimental year, mixture type and study site on corrected relative yields (RY_C_) of grass, chicory and clover using (**a**) unfertilized (N0) and (**b**) fertilized (N1) non-leguminous reference stands.

Effect	(a) N0						(b) N1			
	RY_C_ grass		RY_C_ chicory		RY_C_ clover		RY_C_ grass		RY_C_ chicory	
	*F*	*P*	*F*	*P*	*F*	*P*	*F*	*P*	*F*	*P*
Population	2.4	0.026	–	–	8.5	< 0.001	2.7	0.013	–	–
Year	464.3	< 0.001	109.4	< 0.001	365.1	< 0.001	309.7	< 0.001	175.5	< 0.001
Mixtype	68.3	< 0.001	10.7	0.001	27.3	< 0.001	89.9	< 0.001	8.7	0.004
Site	18.1	0.005	1.6	0.248	0.8	0.403	0.0	0.890	10.1	0.019
Population × Year	2.9	< 0.001	–	–	–	–	2.4	< 0.001	–	–
Year × Mixtype	52.5	< 0.001	23.2	< 0.001	31.4	< 0.001	35.8	< 0.001	24.0	< 0.001
Year × Site	79.5	< 0.001	66.0	< 0.001	40.4	< 0.001	26.5	< 0.001	10.0	< 0.001
Mixtype × Site	45.6	< 0.001	15.7	< 0.001	9.2	< 0.001	50.5	< 0.001	12.6	< 0.001
Year × Mixtype × Site	25.2	< 0.001	5.4	< 0.001	3.8	< 0.001	13.7	< 0.001	6.5	< 0.001

*F*- and *P*-values of sequential Wald chi-square tests are given. – Effect not retained in the minimum adequate model.

**Figure 3:**
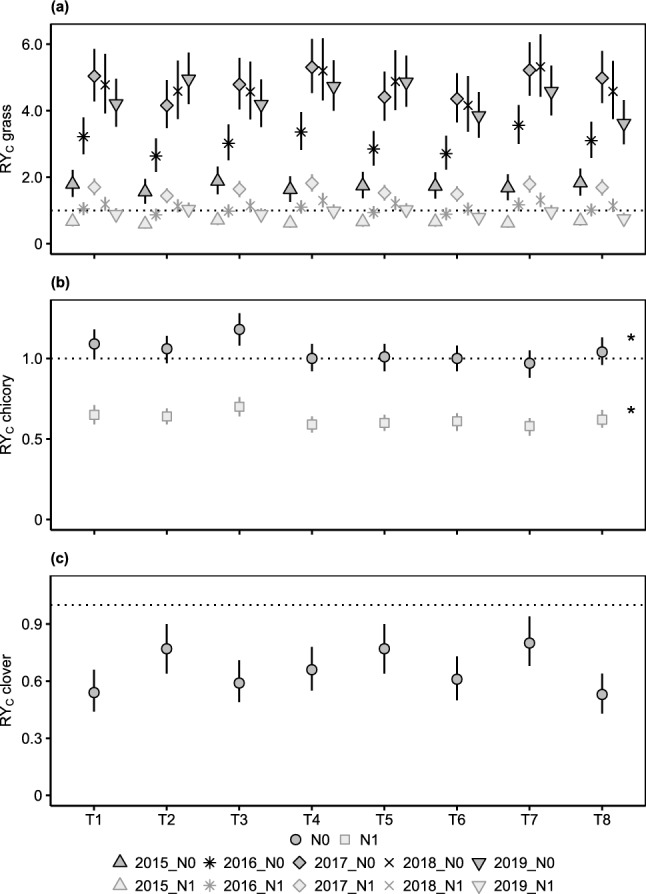
Corrected relative yields (RY_C_) of (**a**) grass, (**b**) chicory and (**c**) clover for different white clover populations (T1–T8) using either unfertilized (N0) or fertilized (N1) non-leguminous pure stands as a reference. For grass RY_C_, the population × year interaction is shown (*n* = 16). Otherwise, the population main effect is presented (chicory RY_C_ *n* = 80, clover RY_C_ *n* = 120). Except for *, given values are model predictions with 95% confidence intervals. Asterisks (*) indicate that arithmetic means with standard errors are shown, as the minimum adequate model did not include the effect of population. Note that scales of the y-axis differ between panels. Pure stand and mixture yield of the white clover populations can be found in Supplementary Table S4.

All white clover populations led to higher observed than expected grass yield (RY_C_ > 1) in any experimental year (Fig. 3a), whereas their clover RY_C_ was consistently below one (Fig. 3c) and their chicory RY_C_ mostly equalled one (Fig. 3b). Despite a significant population × year interaction (*P* < 0.001), grass RY_C_ was generally lowest during the first experimental years (2015–2016).

**Figure 4:**
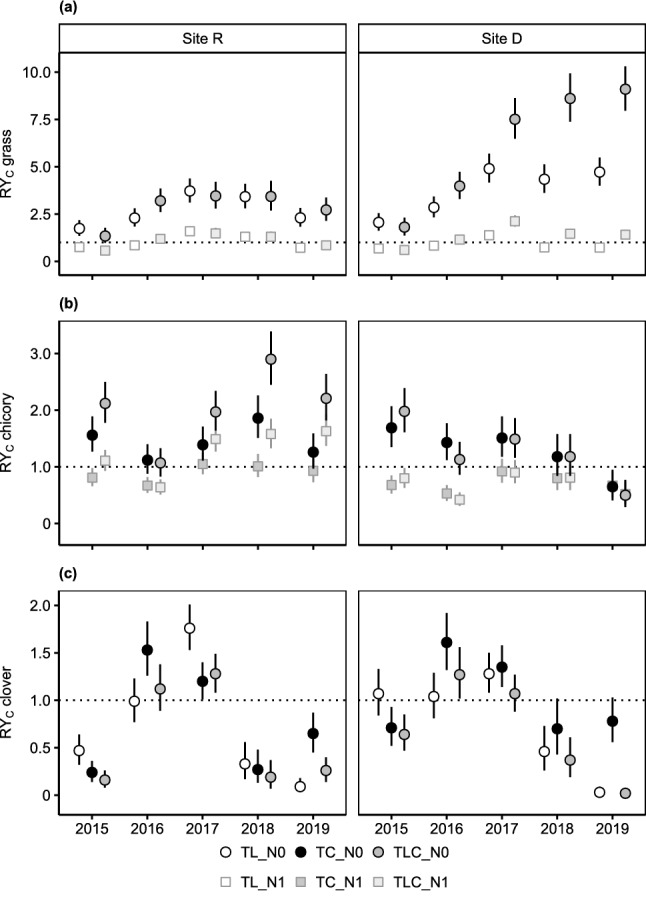
Temporal trends of corrected relative yields (RY_C_) of (**a**) grass, (**b**) chicory and (**c**) clover for different mixture types (TL: clover-grass mixture, TC: clover-chicory mixture, TLC: clover-grass-chicory mixture) at both study sites (site R, site D) using either unfertilized (N0) or fertilized (N1) non-leguminous pure stands as a reference. Given values are model predictions with 95% confidence intervals (*n* = 64). Note that scales of the y-axis differ between panels. Pure stand and mixture yield of grass, chicory and clover can be found in Supplementary Table S4.

Both mixture types accounted for higher observed than expected grass or chicory yield in all or the majority of years, but particularly so the three-species mixture at either site D (grass yield; Fig. 4a) or site R (chicory yield; Fig. 4b). Observed clover yield solely exceeded expectations in 2016 and 2017 (Fig. 4c).

The temporal trends in species RY_C_ were similar for the respective two- and three-species mixtures. While at site D, chicory RY_C_ decreased in the course of the experiment, no clear trend was apparent at site R (Fig. 4b). By contrast, grass RY_C_ increased until 2017 and then levelled off at each site (Fig. 4a).

### Diversity effects of mixture species with fertilized non-leguminous references

Grass and chicory RY_C_ calculated based on fertilized reference stands also depended on the interaction between experimental year, mixture type and study site, and grass RY_C_ was still affected by the interaction of white clover population and experimental year (Table 2b).

For any inspected white clover population, grass and chicory RY_C_ with fertilized reference stands were lower than the corresponding RY_C_ with unfertilized reference stands and the fluctuation of grass RY_C_ over time was weaker (Fig. 3). Additionally, differences between populations in temporal trends of grass RY_C_ diminished with fertilized reference stands.

Moreover, the direction of differences in grass and chicory RY_C_ between experimental years and mixture types was similar between fertilization levels, albeit of smaller extent with fertilized references (Fig. 4).

## Discussion

Our study contributes to the diversity-productivity research by giving further insights into the processes by which diversity effects arise in white-clover-based forage plant mixtures as well as into their driving factors. We show that the choice of the white clover population substantially affects outcome and causes of diversity effects and their temporal trends, though not consistently across all scales. The findings underline the usefulness of a combined analysis of overall (e.g. NE after Loreau and Hector^17^) and individual diversity effects (e.g. RY_C_ after Roscher et al*.*^24^) to gain a better understanding of processes responsible for differential effects of species genotypes.

### Diversity effects of mixtures and their component species

Independent of the chosen white clover population, our study confirms the positive diversity-productivity relationship long known for intensively managed clover-grass mixtures and more recently also described for intensively managed clover-forb or clover-grass-forb mixtures^3,15,26,38^. Overall, all three mixtures turned out to be more productive than expected from the weighted average pure stand productivity for their component species, i.e. there was a positive NE. In line with past research mainly conducted in extensive experimental grassland, the contribution of particular productive species (SE) was smaller in magnitude than that of an overall increased species productivity (CE) and predominantly negative^6,27^. This provides evidence that, also for novel species combinations under more intensive management, interactions between species are more important to account for the generation of a positive NE than disproportionate effects of single species.

Analyses of species behaviour revealed that the contribution of mixture species to positive NE varied but was generally similar between different mixture types. On average, productivity of white clover profited least and not in all experimental years from the cultivation in mixtures. While previous studies have also detected a limited direct contribution of legumes to positive NE, their ability to improve soil nitrogen availability from fixation of atmospheric nitrogen allowed them to contribute indirectly, by enhancing the productivity of their non-leguminous companion species^24,39^. In our study, perennial ryegrass and chicory were overall more productive in mixtures than in pure stands (RY_C_ > 1), and this was less pronounced or even reversed when fertilized instead of unfertilized reference stands were considered. Hence, a facilitative effect of white clover on nitrogen availability for its companion species seems to have been of major importance in our study as well. However, the positive CE with fertilized reference stands and the clover RY_C_ above one in some of the years are indicative of additional, nitrogen-unrelated processes. This corresponds well with results by Marquard et al*.*^22^ or Ergon et al*.*^40^ and is further consistent with findings of positive diversity effects in mixtures without legumes^41^. A more efficient water and light utilization in mixtures, e.g. due to varying root or shoot architecture between mixture species might be the underlying process^40,42^. It is widely accepted that legumes only strongly affect the productivity of their non-leguminous companion species and consequently increase positive diversity effects if nitrogen is the main growth-limiting resource^13,33^. In contrast to perennial ryegrass (and white clover), chicory has the ability to access resources of deeper soil layers, which can reduce its limitation by nitrogen^15^. Smaller average nitrogen deficiencies in unfertilized pure stands of chicory than in those of perennial ryegrass provide evidence that this has been the case in our experiment (see Supplementary Table S4). Hence, this probably explains why the productivity of perennial ryegrass profited notably more than that of chicory from being cultivated in mixtures with white clover, and particularly so at the less fertile study site. It could ultimately explain why the CE of the clover-chicory mixture with unfertilized reference stands was weaker compared to that of mixtures including perennial ryegrass, and differed less strongly from the corresponding value with fertilized reference stands. The overall higher productivity of chicory in mixtures than in pure stands, i.e. overyielding of chicory, more likely resulted to a large extent from complementary use of water and light^41^. Accordingly, overyielding of chicory was stronger at the more fertile and deep-soiled site that enables the realization of varying rooting depths between chicory and its companion species. Remarkably, both the greater overyielding of chicory at the more fertile and deep-soiled site and the greater overyielding of perennial ryegrass at the site with less fertile and shallow soil were notably more pronounced in the three-species than in the two-species mixture.

Results of earlier investigations emphasised that outcome and causes of diversity effects might be altered by the choice of the white clover population. It is common knowledge that intra-specific differences in yield potential, competitive ability, complementarity or nitrogen fixing capacity exist for white clover^35–37^. Thus, Annicchiarico^43^ observed that white clover genotypes differently affect the productivity of their pure stands and mixtures with grasses. Moreover, Zuppinger-Dingley et al*.*^44^ showed that the NE of legume-grass mixtures is smaller with legume and grass populations that were previously cultivated as pure stands than with those previously grown together in mixtures and, hence, displaying increased niche differentiation. In the present study, CE and SE varied between white clover populations, partly confirming our first hypothesis that diversity effects depend on the white clover population. Additional analyses (see Supplementary Table S4) revealed that this was the outcome of stronger biomass yield differences between white clover populations in mixtures than in pure stands. However, the variation of CE and SE between populations was small and cancelled each other out, resulting in a non-significant effect of white clover population on NE. In line with the low productivity benefits by cultivating chicory in mixtures with white clover (see above), solely population-dependent differences in relative mixture productivity (RY_C_) of white clover and perennial ryegrass contributed to the variation of CE and SE. These results agree with findings of increased positive diversity effects by species genotype choice for arable crops^45^. Yet, the impact of genetic identity on diversity effects we found was weaker than that observed for arable crops. This might be associated with the rather small intra-specific differences in morphological, phenological or biochemical characteristics in the present experiment^23,46^. Small differences in those characteristics are assumed to less likely increase the strength of SE than CE^45^. Accordingly, in our study, the choice of the white clover population affected CE more strongly than SE.

The reduced differences in diversity effects between white clover populations when considering fertilized instead of unfertilized reference stands support our third hypothesis that a varying influence of the populations on soil nitrogen availability was of importance to generate these differences. This is further underlined by the finding that average nitrogen deficiency of the three mixture types changed with the white clover population (see Supplementary Table S4).

### Temporal trends in diversity effects of mixtures and their component species

The size of diversity effects turned out to be highly dynamic. Contrary to most previous studies, however, NE did not generally strengthen with time and was not increasingly driven by CE^4,22,27^. Instead, NE fluctuated due to an antagonistic development of CE and SE. This was caused by a changing direction and magnitude of species responses to cultivation in mixture. The generally shared pattern across different mixture types point to a dominant role of varying abiotic conditions^47^. Overall, NE initially grew in strength, especially so for mixtures including perennial ryegrass, and mainly due to increasing overyielding of perennial ryegrass. Hence, it is probably associated with a depletion of soil nitrogen in unfertilized non-leguminous reference stands^47,48^. Allocation of nutrients to the aboveground biomass and its harvest gradually remove nutrients from the soil^42,49^. Furthermore, an absence of regular maintenance efforts such as tillage successively reduces mineralisation of nutrients in the soil^50^. Thus, the ability of white clover to improve soil nitrogen availability can be expected to become increasingly important over time. However, the fact that relative mixture productivity of white clover also increased during the first experimental years indicate additional underlying processes, e.g. the time needed to develop dissimilar root or shoot architectures between mixture species, which allow for a more efficient water and light utilization^27^.

In later years of the experiment, the strength of NE decreased again. One likely cause is the low precipitation in these years. A smaller NE under reduced water availability has also been detected earlier^39^. It was related to a switch of the major growth-limiting resource from nitrogen to water as well as to negative effects of low water availability on nitrogen fixation^13,49^. However, in our study, the magnitude of perennial ryegrass overyielding remained relatively constant during the last years of the experiment and the decline in NE was mainly driven by a reduction in relative productivity of white clover in mixture. A better performance of its neighbours in mixtures compared to pure stands and the resulting stronger depletion of resources might explain why productivity of the drought-sensitive species white clover decreased more strongly in mixtures than in pure stands^31,46^.

Following the arguments brought up in the former sub-section, we expected changes in diversity effects over time to differ between white clover populations (hypothesis 2) and that this at least partly results from their varying impact on soil nitrogen availability (hypothesis 3). If an unequal influence on soil nitrogen availability should serve as a reasonable explanation for dissimilar temporal trends in diversity effects between white clover populations, differences in diversity effects between populations have to increase over time when considering unfertilized reference stands, while not (or at least less) with fertilized reference stands. This is grounded on the assumption that varying effects on soil nitrogen availability gradually increase differences in soil nitrogen content.

Partly in line with our second hypothesis, the choice of the white clover population slightly affected fluctuations in grass RY_C_, though not those in chicory or clover RY_C_, nor in CE, SE and NE. The largest differences between populations in grass RY_C_ were detected in the last experimental year with both unfertilized and fertilized reference stands. However, populations differed less strongly with fertilized reference stands, thereby providing further support for our third hypothesis.

### Agronomic relevance

In European farming practice, white clover is commonly cultivated in mixtures with grasses^36^ using cultivars that are often, for instance in Germany, solely selected based on their performance in pure stands^51,52^.

Earlier work has emphasised the potential of mixture-based breeding programmes for white clover to improve the productivity of mixtures including white clover^43,44^. Our findings of varying clover RY_C_ between white clover populations provide further evidence for that potential, as they indicate that the populations differently affect productivity in pure stands and mixtures. Yet it remained unclear how this could be translated into enhanced mixture yield benefits. Strategic use of intra-specific differences in and further adjustment of white clover characteristics related with the fixed nitrogen amount might help to enhance productivity of white-clover-based mixtures compared to their component species grown as pure stands. This is a prerequisite for farmers to consider the cultivation of more diverse crop stands and, hence, of more sustainable forage cropping systems^25^. However, based on the similar impact of white clover populations on diversity effects across different mixture types in our experiment, there seems to be no potential for mixture-partner-specific breeding of white clover, which was also considered previously^53,54^.

Moreover, the inclusion of non-leguminous forbs like chicory into clover-grass mixtures is currently tested as a means to further improve mixture productivity, especially under dry conditions^16,55^. Stronger diversity effects of the clover-grass-chicory mixture and its grass and clover components at least in years with low precipitation provide evidence for its advantage over the clover-grass mixture, particularly with regard to future climate scenarios.

## Conclusions

Our study reveals that diversity effects and their temporal dynamics differ between white clover populations with varying genetic identity. Concluding from our results, this at least partly resulted from population-dependent differences in the impact on soil nitrogen availability. Independently of the white clover population, our findings confirm positive effects on productivity by cultivating forage species in mixtures and the ability of white clover to improve availability of nitrogen in the soil as an important underlying process. However, the latter turned out to be insufficient as the sole cause and to notably differ in its relevance between the companion species perennial ryegrass and chicory as well as between environmental conditions.

Mixture-based breeding of white clover, targeted towards improving characteristics related with the fixed nitrogen amount might, thus, help to establish more productive agricultural grassland. Future efforts should shed more light on the role of the genetic identity of other forage species for the outcome and causes of diversity effects.

## Materials and methods

### Study sites

The study was conducted on two experimental stations of the University of Göttingen, Germany: Reinshof (site R; 51°29’N, 9°55’E, 157 m a.s.l.) and Deppoldshausen (site D; 51°34’S, 9°58’E, 342 m a.s.l). The soil of site R is a Gleyic Fluvisol with 21% clay, 68% silt and 11% sand in the topsoil and has a depth of 100 cm. Extractable soil nutrient concentrations were 64 mg kg^−1^ phosphorus, 139 mg kg^−1^ potassium (CAL extraction) and 210 mg kg^−1^ magnesium (CaCl_2_ extraction). Soil pH (CaCl_2_) was 6.8. The soil of site D is 40 cm deep and was classified as a Calcaric Leptosol consisting of 34% clay, 55% silt and 2% sand in the topsoil. Extractable soil nutrient concentrations were 38 mg kg^−1^ phosphorus, 133 mg kg^−1^ potassium and 320 mg kg^−1^ magnesium. Soil pH was 7.4.

During data collection (2015–2019), average annual temperature and precipitation were 10.1 °C and 580 mm at site R and 9.5 °C and 593 mm at site D (recorded by on-site weather stations). Weather conditions in each experimental year are given in Supplementary Table S1.

### Experimental design

Eight white clover populations varying in yield potential, phenology and morphology were selected from an ongoing breeding programme for mixed cropping (Deutsche Saatveredelung AG (DSV), Asendorf, Germany). A list with characteristics of the populations can be found in the Supplementary (Table S2). Each population was sown in pure stands, two-species mixtures with perennial ryegrass (‘ELP 060687’) or chicory (‘Puna II’) and in three-species mixtures with perennial ryegrass and chicory. These stands received no nitrogen fertilizer. In addition, perennial ryegrass and chicory were cultivated in pure stands at two levels of nitrogen fertilization, 0 and 240 kg ha^−1^ a^−1^. Fertilizer (NH_4_NO_3_) was applied in four doses per year (80, 60, 60 and 40 kg N ha^−1^), prior to each regrowth cycle. Seeds of perennial ryegrass were provided by DSV and those of chicory were purchased from PGG Wrightson Seeds Ltd (Christchurch, New Zealand). Sowing density was 1000 seeds m^-2^. In mixtures, species were sown at ratios of 0.4 : 0.6 (white clover : perennial ryegrass, white clover : chicory) and 0.4 : 0.3 : 0.3 (white clover : perennial ryegrass : chicory).

Stands were established in summer 2014 on 3 m × 5 m plots. A randomized complete block design with four replications (blocks) at each site was chosen. Pure stands and the three mixtures of each of the eight white clover populations were represented once per block. Each block further comprised one plot with unfertilized and fertilized chicory pure stands as well as two plots with unfertilized and fertilized perennial ryegrass pure stands each, resulting in 38 plots per block. In total, there were 152 plots per site.

Our experiment was performed in accordance with all relevant institutional, national, and international guidelines and regulations for experimental research and field studies on plants/plant materials.

### Aboveground biomass yield

The aboveground biomass of all plots was harvested four times per year over five years from 2015 until 2019. Harvests were carried out in six-week intervals from mid-May to mid-October.

At each harvest, the biomass from a 1.4 m × 5 m area of the plots was cut at 5 cm above ground level and weighed using a combine harvester. A subsample of 250 g fresh matter per plot was oven-dried and weighed to determine the dry matter content of the harvested biomass, which was used to quantify total dry matter yield. Another 250 g subsample was separated manually into white clover, perennial ryegrass, chicory and non-sown species. Species fractions were oven-dried and weighed to estimate the species proportions in total dry matter yield and to calculate species dry matter yield. Total and species dry matter yield were accumulated for each year (see Supplementary Table S4). Mean values of the two replicates of perennial ryegrass pure stands per block were used in this calculation.

### Diversity effects

For each mixture plot, we determined diversity effects of mixtures by calculating the annual net biodiversity effect (NE, Eq. 1) and its components complementarity effect (CE, Eq. 2) and selection effect (SE, Eq. 3) as described by Loreau and Hector^17^. All calculations were done based on both unfertilized (N0) and fertilized (N1) non-leguminous pure stands:

1 $$NE = Y_{O} - Y_{E} = CE + SE$$

2 $$CE = N \times \overline{{\Delta RY_{l} }} \times \overline{{M_{l} }}$$

3 $$SE = N \times cov\left( {\Delta RY_{i} , M_{i} } \right)$$

where *N* is the number of species in the mixture, *M*_*i*_ is the yield of species *i* in pure stand averaged across blocks, *ΔRY*_*i*_ is the deviation of the observed (*RY*_*O*_) from the expected relative yield (*RY*_*E*_) of species *i* in the mixture, with *RY*_*O*_ being the quotient of the yield of species *i* in the mixture and its yield in pure stand within the same block, and *RY*_*E*_ being the sowing proportion of species *i* in the mixture. $\overline{{\Delta RY }_{l}}$ and $\overline{{M }_{l}}$ are the mean *ΔRY*_*i*_ and *M*_*i*_ of all species in the mixture. *Y*_*O*_ and *Y*_*E*_ are the sum of the observed and of the expected yield of all species in the mixture, with the expected yield of species *i* being the product of *RY*_*E*_ and *M*_*i*_ (‘Pure stand’ refers to ‘monoculture’ in the original description of indices). The NE is positive when the yield of a mixture is higher than expected based on the weighted average (by sowing proportion of species in the mixture) of the pure stand yields for the component species. The CE is positive when species yields in the mixture are on average higher than expected based of the weighted average pure stand yield of the species and is negative when the reverse is true. The SE is positive when species with higher-than-average pure stand yield dominate the mixture and is negative when the same applies to species with lower-than-average pure stand yield.

In accordance with the approach of Roscher et al*.*^24^, we also assessed diversity effects of mixture species by computing corrected relative yields for each mixture species (RY_C_, Eq. 4). This was done separately for both fertilization levels of non-leguminous pure stands:

4 $${RY}_{C}=\frac{{RY}_{O}}{{RY}_{E}}$$

where *RY*_*O*_ is the observed relative yield of species *i* in the mixture and *RY*_*E*_ is its expected relative yield. The *RY*_*C*_ is > 1 when the yield of species *i* in the mixture is higher than expected based on its yield in pure stand after accounting for different sowing proportions and is < 1 when the reverse is true.

### Statistical analysis

The statistical analysis of the data was conducted in the R software environment version 4.0.3 using linear mixed effects models of the ‘nlme’ software package^56^.

To analyse diversity effects, we modelled NE, CE, SE and RY_C_ for both fertilization levels as a function of white clover population, experimental year, mixture type, study site and all their interactions as fixed effects. Plot nested in block were included as random effects in the models. For each full model, homogeneity of variance and normality of the residuals were checked visually. To fulfil these model assumptions, RY_C_ were square-root transformed and appropriate variance structures were added to the models where needed (see Supplementary Table S3). The adjusted full models were simplified based on the second order Akaike information criterion corrected for small sample sizes (AICc) as implemented in the ‘MuMIn’ software package^57^. Sequential Wald chi-square tests were used to test the significance of the fixed effects remaining in the final models. A significance level of *α* < 0.05 was chosen throughout.

## Supplementary Information

Supplementary Tables.

## Data Availability

The datasets generated during and/or analysed during the current study are available in the Zenodo repository, 10.5281/zenodo.5806738 (Nölke, Isabelle, Tonn, Bettina, Komainda, Martin, Heshmati, Sara & Isselstein, Johannes. (2022). Dataset accompanying Nölke et al. 2022. The choice of the white clover population alters overyielding of mixtures with perennial ryegrass and chicory and underlying processes. Scientific Reports. Zenodo).
