# Supplementary material for: The choice of the white clover population alters overyielding of mixtures with perennial ryegrass and chicory and underlying processes
Source: Sci Rep. 2022 Jan 21;12:1155. doi: 10.1038/s41598-022-05100-6 (PMC8782889; doi:10.1038/s41598-022-05100-6)
Supplement: Supplementary file 1 — Supplementary Tables. [file 41598_2022_5100_MOESM1_ESM.pdf]

Supplementary information

The choice of the white clover population alters overyielding of mixtures with perennial ryegrass and chicory and underlying processes

Isabelle Nölke[1,2,*], Bettina Tonn[1,2,3], Martin Komainda[1], Sara Heshmati[1,2,4]& Johannes Isselstein[1,2]

[1] Division of Grassland Science, Department of Crop Sciences, University of Göttingen, Von-Siebold-Str. 8, 37075 Göttingen, Germany
[2] Centre of Biodiversity and Sustainable Land Use (CBL), University of Göttingen, Büsgenweg 1, 37077 Göttingen, Germany
[3] Present address: Department of Livestock Sciences, Research Institute of Organic Agriculture (FiBL), Ackerstrasse 113, 5070 Frick, Switzerland
[4] Present address: Plant Ecology Group, Institute of Ecology and Evolution, University of Tübingen, Auf der Morgenstelle 5, 72076 Tübingen, Germany
[*] isabelle.noelke@uni-goettingen.de

	Temperature		Precipitation	
	Site R	Site D	Site R	Site D
2015	10.1	9.7	700	633
2016	9.7	9.0	543	548
2017	9.8	9.0	695	791
2018	10.4	10.0	416	444
2019	10.3	9.7	546	550

Table S1. Annual mean temperature (°C) and total precipitation (mm) at both study sites (site R, site D) during experimental years (2015–2019) recorded by on-site weather stations.

Characteristic	Population							
	T1	T2	T3	T4	T5	T6	T7	T8
Leaf size	small to medium	large	medium	medium to large	medium to large	medium	medium	medium to large
Flowering	early	interme-diate	interme-diate	late	late	very late	early	early to interme-diate
Flowering intensity	good	medium	medium	medium	medium	high	medium	very high
Yield in mixture	moderate	high	high	low in Germany/ high in France	high	low	high	medium

Table S2. Description of utilized white clover populations. White clover populations are from an ongoing breeding programme for mixed cropping by the breeding company DSV (Deutsche Saatveredelung AG, Asendorf, Germany). Their specific characteristics were recorded in multi-site field trails prior to the study.

Model		Variance structure
N0	CE	varComb(varIdent(form = ~ 1|mixtype), varIdent(form = ~ 1|year))
	SE	varComb(varIdent(form = ~ 1|mixtype), varIdent(form = ~ 1|year))
	NE	varIdent(form = ~ 1|year)
N1	CE	–
	SE	varComb(varIdent(form = ~ 1|mixtype), varIdent(form = ~ 1|site))
	NE	varComb(varIdent(form = ~ 1|mixtype), varIdent(form = ~ 1|year))
N0	RY_C grass	varComb(varIdent(form = ~ 1|mixtype), varIdent(form = ~ 1|year))
	RY_C chicory	varComb(varIdent(form = ~ 1|year), varIdent(form = ~ 1|site))
	RY_C clover	varIdent(form = ~ 1|year)
N1	RY_C grass	varComb(varIdent(form = ~ 1|mixtype), varIdent(form = ~ 1|site))
	RY_C chicory	varComb(varIdent(form = ~ 1|year), varIdent(form = ~ 1|site))

Table S3. Variance structures fitted to linear mixed effect models to analyse effects on complementarity effect (CE), selection effect (SE) and net biodiversity effect (NE) as well as on corrected relative yields (RY_C) of grass, chicory and clover using either unfertilized (N0) or fertilized (N1) non-leguminous reference stands. – no variance structure fitted.

	Total yield	Clover yield	Grass yield	Chicory yield	Nitrogen yield	NNI
T1	4720.0 ± 2249.6	4044.1 ± 2555.2			210.9 ± 53.3	1.32 ± 0.17
T2	5226.6 ± 2565.9	4971.6 ± 2757.2			223.1 ± 79.8	1.33 ± 0.24
T3	4637.6 ± 2381.6	4048.5 ± 2612.2			193.8 ± 55.6	1.21 ± 0.20
T4	4839.6 ± 2463.3	4359.9 ± 2708.4			215.7 ± 65.3	1.33 ± 0.18
T5	5182.0 ± 2618.5	4955.7 ± 2772.8			231.4 ± 68.2	1.34 ± 0.21
T6	4656.4 ± 2342.7	3821.1 ± 2712.3			200.3 ± 59.0	1.24 ± 0.21
T7	5468.5 ± 2664.4	5113.0 ± 2866.5			249.4 ± 64.9	1.41 ± 0.19
T8	4615.5 ± 2261.1	3973.9 ± 2513.9			204.2 ± 52.5	1.28 ± 0.15
L_N0	2235.1 ± 1320.4		2137.6 ± 1339.2		49.7 ± 23.2	0.50 ± 0.11
L_N1	6596.5 ± 2739.0		6563.2 ± 2747.0		151.7 ± 60.0	0.85 ± 0.14
C_N0	5966.9 ± 2649.2			5534.1 ± 2703.8	128.9 ± 50.4	0.75 ± 0.13
C_N1	10856.7 ± 4446.6			9831.7 ± 5317.4	260.1 ± 84.5	1.03 ± 0.17
T1_L	5397.4 ± 2527.0	1581.7 ± 1657.2	3773.9 ± 1293.4		153.6 ± 75.1	0.89 ± 0.26
T2_L	5664.9 ± 2520.0	2219.3 ± 2159.5	3406.5 ± 1118.2		177.6 ± 80.6	0.99 ± 0.27
T3_L	5125.7 ± 2274.8	1523.7 ± 1606.3	3569.2 ± 1203.5		147.7 ± 64.6	0.88 ± 0.25
T4_L	5532.3 ± 2541.0	1762.9 ± 1819.3	3752.4 ± 1244.2		166.4 ± 77.1	0.94 ± 0.25
T5_L	5580.9 ± 2437.8	1966.8 ± 1920.6	3581.9 ± 1216.6		168.0 ± 76.4	0.96 ± 0.26
T6_L	4892.9 ± 2523.5	1592.0 ± 1740.8	3267.4 ± 1239.9		150.5 ± 69.1	0.89 ± 0.25
T7_L	5947.7 ± 2709.6	2244.8 ± 2111.4	3686.4 ± 1181.9		183.2 ± 81.3	1.00 ± 0.27
T8_L	4852.5 ± 2341.6	1477.0 ± 1458.3	3352.3 ± 1171.1		150.1 ± 58.9	0.91 ± 0.20
T1_C	7371.0 ± 2796.2	1643.2 ± 1417.9		5227.0 ± 2790.2	186.4 ± 59.6	0.94 ± 0.19
T2_C	7842.2 ± 2901.8	2352.7 ± 2060.9		5011.4 ± 2624.3	208.9 ± 60.1	1.01 ± 0.19
T3_C	7315.0 ± 2586.5	1698.5 ± 1681.7		5000.4 ± 2564.4	182.9 ± 52.7	0.93 ± 0.18
T4_C	6794.8 ± 2429.0	1603.6 ± 1542.1		4729.3 ± 2409.7	169.9 ± 56.6	0.91 ± 0.20
T5_C	7071.7 ± 3045.9	2301.2 ± 1726.9		4379.5 ± 2597.0	197.1 ± 69.4	1.01 ± 0.21
T6_C	6708.6 ± 2508.3	1557.4 ± 1489.6		4686.9 ± 2329.1	165.8 ± 50.0	0.89 ± 0.17
T7_C	7368.0 ± 3083.3	2643.9 ± 1831.8		4438.1 ± 3023.3	208.2 ± 59.0	1.05 ± 0.20
T8_C	6956.9 ± 3023.7	1336.2 ± 1216.3		5157.2 ± 2868.1	174.3 ± 67.8	0.90 ± 0.19
T1_LC	6326.1 ± 2386.1	1013.3 ± 1084.7	2143.7 ± 855.4	3162.7 ± 2198.6	149.7 ± 45.3	0.84 ± 0.17
T2_LC	6901.4 ± 2529.6	1839.6 ± 1811.4	2034.2 ± 835.9	3007.2 ± 1905.4	176.2 ± 68.3	0.93 ± 0.23
T3_LC	7202.5 ± 2810.4	1327.4 ± 1433.7	2172.7 ± 676.8	3659.2 ± 2540.7	174.3 ± 58.8	0.89 ± 0.18
T4_LC	6828.4 ± 2397.1	1553.9 ± 1610.3	2365.5 ± 835.6	2884.6 ± 1991.9	166.3 ± 64.4	0.95 ± 0.24
T5_LC	7448.3 ± 2791.8	2079.0 ± 1861.2	2249.1 ± 889.1	3094.0 ± 2045.6	190.5 ± 74.3	0.91 ± 0.22
T6_LC	6073.5 ± 2278.2	1343.3 ± 1512.3	1929.3 ± 741.3	2789.5 ± 1749.7	148.8 ± 61.5	0.85 ± 0.21
T7_LC	7383.4 ± 2946.0	1938.8 ± 1866.6	2463.7 ± 789.7	2972.1 ± 2072.4	192.6 ± 77.8	0.96 ± 0.25
T8_LC	6360.2 ± 2462.0	1086.8 ± 1113.2	2223.3 ± 883.0	3044.2 ± 2228.9	153.0 ± 47.5	0.84 ± 0.16

Table S4. Mean values (± standard deviation) of total and species annual biomass yield (kg ha^{-1}), annual nitrogen yield (kg ha^{-1}) and annual nitrogen nutrition index (NNI) for white clover populations (T1–T8), perennial ryegrass (L) and chicory (C) in pure stands and mixtures across experimental years and study sites (n = 40 (80 for L)). Pure stands of perennial ryegrass and chicory were either unfertilized (N0) or nitrogen-fertilized (N1). Annual nitrogen yield = Annual weighted average nitrogen content × Annual total biomass yield; NNI = Annual weighted average nitrogen content/(4.8 × Annual total biomass yield$^{-0.34}$), with NNI < 1 nitrogen deficit and NNI > 1 nitrogen excess. Nitrogen content was determined with near infrared reflectance spectroscopy (NIRS) of milled subsamples of the harvested biomass.
